# Targeted depletion of dysfunctional hematopoietic stem cells mitigates myeloid-biased differentiation in aged mice

**DOI:** 10.1038/s41421-025-00810-3

**Published:** 2025-06-10

**Authors:** Xiangle Ren, Yuting Wang, Yi Zhang

**Affiliations:** 1 Howard Hughes Medical Institute, Boston Children’s Hospital, Boston, MA USA; 2 Program in Cellular and Molecular Medicine, Boston Children’s Hospital, Boston, MA USA; 3 Division of Hematology/Oncology, Department of Pediatrics, Boston Children’s Hospital, Boston, MA USA; 4 Department of Genetics, Harvard Medical School, Boston, MA USA; 5 Harvard Stem Cell Institute, WAB-149G, Boston, MA USA

**Keywords:** Ageing, Haematopoietic stem cells

Dear Editor,

Hematopoietic stem cells (HSCs) reside at the apex of the blood hierarchy and play important roles throughout an organism’s lifespan in adapting to physiological demands^1^. However, aging leads to a decline in HSC function, characterized by the accumulation of dysfunctional HSCs and skewed differentiation towards the myeloid lineage^1^. This shift promotes the production of pro-inflammatory myeloid cells at the expense of lymphoid cells^2^, contributing to immunological deficits, including decreased adaptive immune competence and increased myeloid malignancy and myeloproliferative diseases^3^.

We have recently demonstrated the presence of molecularly and functionally heterogeneous LT-HSC (long-term HSC, referred to as HSC) populations in aged mice, including a functionally “younger” population and a functionally defective “older” population distinguished by the levels of CD150 expression^4^. Compared to the CD150^low^ HSCs, the CD150^high^ HSCs from old mice exhibit an older transcriptome, chromatin, epi-age, as well as differentiation defects. Transplantation experiments demonstrated that mice that received CD150^low^ HSCs exhibit a better hematopoietic system, physical functions, younger epi-age, and longer lifespan compared to those that received CD150^high^ HSCs from the same old donor mouse^4^. The CD150^high^ HSC subset gradually accumulates during aging. Reducing the ratio of CD150^high^ to CD150^low^ HSCs, thereby mimicking the depletion of dysfunctional CD150^high^ HSCs, alleviates aging-related phenotypes in old recipient mice^4^. Others have also shown that CD150^high^ HSCs are intrinsically biased toward myeloid lineage differentiation, which is a key component of hematopoietic aging^5,6^. These findings suggest that targeted removal of the dysfunctional CD150^high^ HSCs from the heterogeneous HSC pool in aged mice under physiological conditions might provide a viable strategy for mitigating aging-related phenotypes.

To evaluate the feasibility of targeting CD150^high^ HSCs, we first examined the expression profiles of CD150 in various cell types and tissues and found that CD150 exhibits the highest expression in HSCs among hematopoietic cell types (Supplementary Fig. S1a). In tissues and organs, CD150 is mainly expressed in the thymus (Supplementary Fig. S1b). The relatively restricted expression makes CD150 an excellent marker for targeted depletion with minimal potential off-target toxicity. To eliminate dysfunctional CD150^high^ HSCs, we used an antibody-toxin conjugation approach. Saporin is a ribosome-inactivating protein that inhibits protein synthesis^7^. We selected Saporin as the payload for the antibody-toxin approach due to its high enzymatic activity, stability, and resistance to conjugation procedures and blood proteases^8^. In addition, Saporin-based immunotoxins have been extensively characterized in preclinical and clinical studies^9^.

We conjugated biotin-labeled CD150 antibody with streptavidin-labeled Saporin. The resulting CD150-SAP complex is expected to induce cell death upon endocytosis into targeted cells (Supplementary Fig. S1c). We first tested the HSC killing effect of the CD150-SAP complex in vitro. Since HSC killing depends on the presence of CD150 on the HSC cell surface, we examined CD150 expression during HSC culturing. Although HSCs rapidly differentiate into downstream multipotent progenitor cells, CD150 expression is still maintained after 3 and 6 days of culturing (Supplementary Fig. S1d), indicating that the system can be used for evaluating the killing efficacy of CD150-SAP. To demonstrate the killing effect, HSCs were purified from old mice and treated with different concentrations of CD150-SAP. The results showed that CD150-SAP treatment resulted in a dose-dependent killing (Supplementary Fig. S1e). Interestingly, we noticed that about 100–1000-fold higher concentrations of IgG-SAP or CD150 Ab were needed to cause a similar effect, indicating that the CD150-SAP complex-mediated killing is relatively specific and efficient. To evaluate whether such a strategy can achieve preferential killing of CD150^high^ HSCs relative to that of CD150^low^ HSCs, we mixed equal numbers of CD150^low^ HSCs and CD150^high^ HSCs purified from CD45.1 or CD45.2 age- and sex-matched 17-month-old mice, respectively (Supplementary Fig. S1f). We found that with the increase in CD150-SAP concentration, the numbers of CD150^low^ and CD150^high^ HSCs were both decreased when compared to the PBS-treated control (Fig. 1a). However, at 0.01 nM, CD150-SAP significantly reduced the number of CD150^high^ HSCs but had no obvious effect on CD150^low^ HSCs (Fig. 1a), indicating that CD150-SAP exhibited a specific killing effect on CD150^high^ HSCs under this condition. In addition, the decreased ratio of CD150^high^ to CD150^low^ HSCs in response to increased CD150-SAP also supports the preferential targeting of CD150^high^ HSCs (Fig. 1b).

**Fig. 1:**
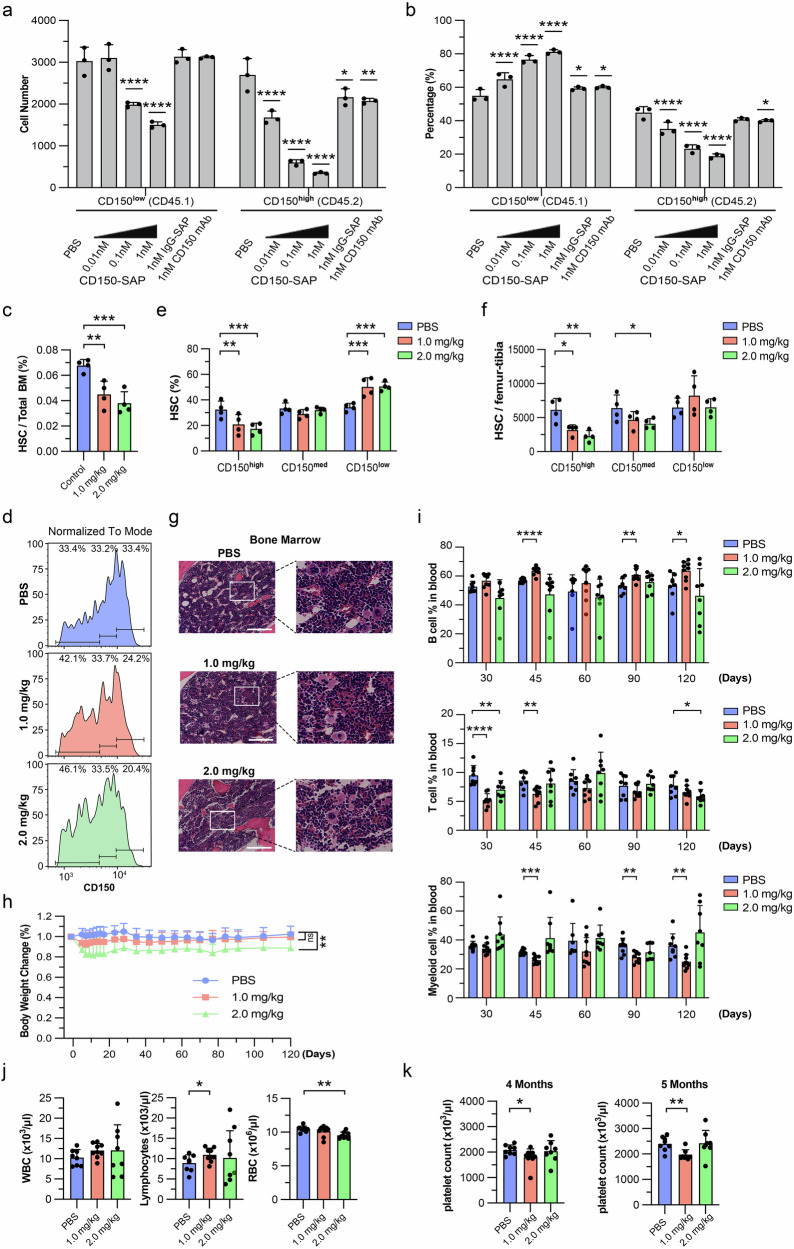
In vivo depletion of the dysfunctional CD150^high^ HSCs reduces myeloid differentiation bias and mitigates aging-associated platelet increase in old mice. **a**, **b** Specific elimination of CD150^high^ HSCs by careful titration of the CD150-SAP complex concentration. The cell number (**a**) and percentage (**b**) of CD45.1^+^ and CD45.2^+^ cells were quantified after 3-day treatment. **c** Percentage of HSCs in the bone marrow 3 weeks post-treatment. **d** Distribution of HSCs based on CD150 expression levels in mice with or without in vivo CD150-SAP treatment. HSCs were evenly divided into 3 groups (CD150^low^, CD150^med^, and CD150^high^) based on CD150 signal intensity in PBS-injected control mice, which served as a reference for CD150 level grouping. **e** Percentages of CD150^high^, CD150^med^, and CD150^low^ HSCs with or without in vivo CD150-SAP treatment. **f** Absolute numbers of CD150^high^, CD150^med^, and CD150^low^ HSCs per femur and tibia with or without CD150-SAP treatment. **g** H&E staining of bone marrow; scale bar, 20 μm. **h** Body weight changes following PBS or CD150-SAP injection. **i** Percentages of B cells, T cells, and myeloid cells in peripheral blood analyzed at different time points post-CD150-SAP treatment. **j** Complete blood count analysis performed 4 months post-CD150-SAP treatment. WBC, white blood cell; RBC, red blood cell. **k** Platelet counts measured at 4 months (left) and 5 months (right) post CD150-SAP treatment. Data are presented as mean ± SD. Statistical significance was determined by One-way ANOVA (**c**, **j** and **k**) or Two-way ANOVA (**a**, **b**, **e**, **f**, **h** and **i**) (*P < 0.05, **P < 0.01, ***P < 0.001, ****P < 0.0001). Mouse number per group: *n* = 4 (**c**–**g**), *n* = 8–9 (**h**–**k**).

Having demonstrated preferential killing of CD150^high^ HSCs by CD150-SAP in vitro, we next asked whether the same is true in vivo. Since CD150 is one of the surface markers used for HSC sorting and FACS analysis, the injected CD150-SAP complex could hinder flow cytometry detection of CD150^+^ cells after CD150-SAP treatment (Supplementary Fig. S2a). To avoid such a masking effect, we performed a time-course experiment to determine the length the injected CD150-SAP complex can persist (Supplementary Fig. S2b). We found that the percentage of labeled HSCs gradually decreases, reaching zero by day 20 (Supplementary Fig. S2c), indicating complete clearance of the injected biotin-CD150 antibody by this time point. Consequently, we established a three-week interval as the minimum time required for downstream analysis to avoid potential antibody masking effects.

Saporin-based immunotoxins have been previously used to deplete target cells in the hematopoietic system, with dosages ranging from 1 to 5 mg/kg^10,11^. We found that mice treated with CD150-SAP at doses above 4 mg/kg experienced noticeable weight loss, likely due to cellular toxicity (data not shown). To minimize the toxicity, we treated a group of 15-month-old mice with lower doses (1 or 2 mg/kg) of CD150-SAP (Supplementary Fig. S2d). The percentage of HSCs in the bone marrow showed a dose-dependent decrease (Fig. 1c), indicating that HSCs were partially depleted by the treatment. To assess whether CD150^high^ HSCs were preferentially targeted, the distribution of HSCs based on their CD150 levels was analyzed, which revealed a dose-dependent shift of the CD150^high^ to the CD150^low^ population (Fig. 1d, e). Furthermore, the absolute number of CD150^high^ HSCs decreased following CD150-SAP treatment, while CD150^low^ HSCs were unaffected, further demonstrating the specificity of the treatment in vivo (Fig. 1f).

To comprehensively characterize the potential off-target effects of CD150-SAP treatment, both the percentages and absolute numbers of hematopoietic progenitor cells and differentiated lineage cells in the bone marrow were analyzed three weeks post-treatment. No obvious effect on multipotent progenitor cell 1 (MPP1), MPP2, or MPP4 (Supplementary Fig. S3a, b, d), but a slight increase in MPP3 cells was observed by the CD150-SAP treatment (Supplementary Fig. S3c). These MPPs give rise to lineage-restricted common lymphocyte progenitor (CLP) and common myeloid progenitor (CMP), which further give rise to granulocyte-monocyte progenitor (GMP), megakaryocyte-erythroid progenitor (MEP) and megakaryocyte progenitor (MkP). Among these more committed progenitors, only MEPs were significantly decreased in absolute numbers after CD150-SAP treatment (Supplementary Fig. S3e–i). The decreased percentages in CMPs, GMPs, MEPs, and MkPs can be accounted for by an overall increase in the bone marrow cellularity following the treatment (Supplementary Fig. S3j). Regarding to the differentiated lineage cells, CD150-SAP treatment increased the total numbers of granulocytes and monocytes, while having no effect on erythroblasts and pro-/pre-B cells (Supplementary Fig. S3k–n). The observed increase in granulocytes and monocytes is consistent with the overall increase in bone marrow cellularity (Supplementary Fig. S3j), as well as the increase in MPP3 cells, which are biased toward granulocyte and monocyte differentiation. Importantly, the comprehensive analysis showed that CD150-SAP treatment did not affect the absolute numbers of MPP2 and MkP cells, despite their low expression levels of CD150 (Supplementary Fig. S1a). Collectively, these results strongly support that CD150-SAP can specifically and preferentially deplete CD150^high^ HSCs in vivo.

To evaluate the potential toxicity of CD150-SAP treatment, histology assessments of major organs and serum biochemical tests were performed. Hematoxylin and eosin (H&E) staining of the bone marrow, spleen, kidney, liver, brain, heart, and lung revealed overall normal morphology three weeks post-CD150-SAP treatment (Fig. 1g; Supplementary Fig. S4a). A reduction in serum levels of the transaminases alanine aminotransferase (ALT) and aspartate aminotransferase (AST) was observed after treatment, indicating no liver toxicity at the administered doses (Supplementary Fig. S4b). Similarly, no evidence of kidney toxicity was detected, as shown by unchanged uric acid levels and decreased urea and creatinine levels in the serum following the treatment (Supplementary Fig. S4b). To further assess systemic toxicity, body weight was monitored post-treatment, which revealed no significant changes in the 1.0 mg/kg CD150-SAP treatment group, and only a slight weight loss in the 2.0 mg/kg CD150-SAP treatment group compared to the PBS controls (Fig. 1h), suggesting no substantial systemic toxicity at the doses administered.

To monitor the effect of these treatments on hematopoiesis, peripheral blood analyses were performed. We found that treatment at 1.0 mg/kg CD150-SAP significantly increased B cell percentages and reduced myeloid cell percentages at days 45, 90, and 120 post-treatments (Fig. 1i). The percentage of T cells decreased at the early time points but rebounded to normal levels following treatment with 1.0 mg/kg CD150-SAP (Fig. 1i). However, those improvements in myeloid differentiation bias were not observed in the 2.0 mg/kg CD150-SAP treatment group, even with a decrease in T cells at day 120 (Fig. 1i), suggesting that at this dose there might be low level of cell toxicity for T cells, consistent with the relatively higher level of CD150 expression in thymus (Supplementary Fig. S1b).

Consistent with the peripheral blood analyses shown above, complete blood count analysis performed 4 months post-treatment revealed that mice treated with 1 mg/kg CD150-SAP had normal white and red blood cell counts and showed an increase in lymphocytes (Fig. 1j), while the 2 mg/kg treatment caused a reduction in red blood cell counts. Previous studies have shown that platelets become hyper-reactive, and the number also increases upon aging in mice^12,13^. Platelet dysregulation plays a major role in cardiovascular disorders and is a leading cause of death in the elderly. To investigate whether depletion of myeloid-biased CD150^high^ HSCs affects platelet generation, we quantified platelet numbers at 4- and 5-months post-treatment. We found that treatment at 1 mg/kg reduced platelet counts at both time points (Fig. 1k), indicating a mitigation of the increased platelet differentiation of aged HSCs. Taken together, these results indicate that depletion of the dysfunctional CD150^high^ HSCs can partially correct the age-associated myeloid- and platelet-biased differentiation, potentially improving hematopoietic differentiation balance.

The development of translatable rejuvenation approaches targeting the hematopoietic system holds great promise for mitigating age-associated blood disorders and the decline in immune function in aged individuals. Although various approaches have been explored to rejuvenate old HSCs and restore their youthful state, many of these methods are non-specific with limited success, and a significant proportion of rejuvenation methods rely on transplantation, which involves irradiation and other manipulations that cannot reflect the situation of physiological conditions^1^. The CD150-SAP strategy described here provides a new avenue for the targeted elimination of the dysfunctional HSCs in older individuals. Our data support improved hematopoietic differentiation after CD150-SAP treatment in vivo. While it remains to be determined whether the improved lineage differentiation output of HSCs will translate into whole body rejuvenation, the observed improvement in hematopoietic differentiation is consistent with findings from a recent study using an antibody cocktail that targets both myeloid-biased HSCs and progenitor cells (c-Kit positive cells) simultaneously^14^. We note that our approach uses a fundamentally different cell elimination mechanism that blocks protein translation by inhibiting the ribosome. In contrast, their method eliminates myeloid-biased HSCs through macrophage-mediated phagocytosis, which involves administering a cocktail of three antibodies — a target antibody (e.g., NEO1, CD62P, or CD150), a c-Kit antibody, and a CD47-blocking antibody to disrupt the “don’t eat me” signal to increase phagocytosis — at relatively high doses (200 µg targeting antibody, 30–100 µg c-Kit antibody, and 2.6 mg CD47 antibody per mouse). The absence of toxicity assessments makes it difficult to evaluate the overall feasibility of systemic rejuvenation using the antibody cocktail approach. On the other hand, the amount of CD150 antibody used in our study (1 mg/kg CD150-SAP) is less than one-tenth of the dosage employed in the antibody cocktail approach, underscoring the efficiency, feasibility, and potential safety of our approach. Consistently, comprehensive analyses of the bone marrow compartment and systemic toxicity in our study revealed high specificity with minimal toxicity following CD150-SAP treatment. Importantly, our approach preferentially eliminates the dysfunctional CD150^high^ HSCs in old mice, while sparing functional CD150^low^ HSCs (Fig. 1f). Given that the aging-associated HSC dysfunction is conserved in humans^15^, our study provides a foundation for possible translational studies in the future.

## Supplementary information

Supplementary Information

## References

[1] Kasbekar, M., Mitchell, C. A., Proven, M. A. & Passegue, E. *Cell Stem Cell* **30**, 1403–1420 (2023). 10.1016/j.stem.2023.09.013 PMC10842631 37865087

[2] Yamamoto, R. & Nakauchi, H. *Mech. Ageing Dev.* **192**, 111378 (2020). 10.1016/j.mad.2020.111378 PMC7686268 33022333

[3] Wahlestedt, M., Pronk, C. J. & Bryder, D. *Stem Cells Transl. Med.* **4**, 186–194 (2015). 10.5966/sctm.2014-0132 PMC4303356 25548388

[4] Wang, Y. et al. *Cell Res.* **35**, 45–58 (2025). 10.1038/s41422-024-01057-5 PMC11701126 39743633

[5] Beerman, I. et al. *Proc. Natl. Acad. Sci. USA* **107**, 5465–5470 (2010).

[6] Morita, Y., Ema, H. & Nakauchi, H. *J. Exp. Med.* **207**, 1173–1182 (2010). 10.1084/jem.20091318 PMC2882827 20421392

[7] Ippoliti, R., Lendaro, E., Bellelli, A. & Brunori, M. *FEBS Lett.* **298**, 145–148 (1992). 10.1016/0014-5793(92)80042-f 1544437

[8] Polito, L., Bortolotti, M., Mercatelli, D., Battelli, M. G. & Bolognesi, A. *Toxins* **5**, 1698–1722 (2013). 10.3390/toxins5101698 PMC3813907 24105401

[9] Giansanti, F., Flavell, D. J., Angelucci, F., Fabbrini, M. S. & Ippoliti, R. *To**xins* **10**, 82–114 (2018). 10.3390/toxins10020082 PMC5848183 29438358

[10] Palchaudhuri, R. et al. *Nat. Biotechnol.* **34**, 738–745 (2016). 10.1038/nbt.3584 PMC5179034 27272386

[11] Czechowicz, A. et al. *Nat. Commun.* **10**, 617 (2019). 10.1038/s41467-018-08201-x PMC6365495 30728354

[12] Dayal, S. et al. *Circulation* **127**, 1308–1316 (2013). 10.1161/CIRCULATIONAHA.112.000966 PMC4447136 23426106

[13] Poscablo, D. M. et al. *Cell* **187**, 3090–3107.e21 (2024). 10.1016/j.cell.2024.04.018 PMC12047039 38749423

[14] Ross, J. B. et al. *Nature* **628**, 162–170 (2024). 10.1038/s41586-024-07238-x PMC11870232 38538791

[15] Geiger, H., de Haan, G. & Florian, M. C. *Nat. Rev. Immunol.* **13**, 376–389 (2013). 10.1038/nri3433 23584423

