# Supplementary material for: Targeted depletion of dysfunctional hematopoietic stem cells mitigates myeloid-biased differentiation in aged mice
Source: Cell Discov. 2025 Jun 10;11:56. doi: 10.1038/s41421-025-00810-3 (PMC12149288; doi:10.1038/s41421-025-00810-3)
Supplement: Supplementary file 1 — Supplementary Information [file 41421_2025_810_MOESM1_ESM.pdf]

Supplementary Information for

Targeted depletion of dysfunctional hematopoietic stem cells mitigates myeloid-biased differentiation in aged mice

Xiangle Ren[1,2,3], Yuting Wang[1,2,3], Yi Zhang[1,2,3,4,5*]

[1]Howard Hughes Medical Institute, Boston Children's Hospital, Boston, Massachusetts 02115, USA;

[2]Program in Cellular and Molecular Medicine, Boston Children's Hospital, Boston, Massachusetts 02115, USA;

[3]Division of Hematology/Oncology, Department of Pediatrics, Boston Children's Hospital, Boston, Massachusetts 02115, USA;

[4]Department of Genetics, Harvard Medical School, Boston, Massachusetts 02115, USA;

[5]Harvard Stem Cell Institute, WAB-149G, 200 Longwood Avenue, Boston, Massachusetts 02115, USA.

[*] corresponding author: Yi Zhang

E-mail: yzhang@genetics.med.harvard.edu

This PDF file includes:

- Materials and Methods
- References
- Supplementary figures

Material and Methods

Mice

All experiments were conducted in accordance with the National Institute of Health Guide for Care and Use of Laboratory Animals and approved by the Institutional Animal Care and Use Committee (IACUC) of Boston Children's Hospital and Harvard Medical School. For in vitro CD150-SAP experiments, 17 to 20-month-old C57BL/6 mice (Jackson Lab #000664) and B6.SJL-*Ptprc*^{a} *Pepc*^{b}/BoyJ mice (Jackson Lab #002014) were used. For biotin-CD150 labeling experiments, 8-week-old C57BL/6 mice (Jackson Lab #000664) were used. For in vivo CD150-SAP depletion experiments, 15-month-old C57BL/6 mice (Jackson Lab #000664) were used.

Antibody-Saporin conjugation and administration

Biotin-CD150 (Biolegend, clone TC15-12F12.2, catalog # 115908) or biotin-IgG (BioLegend, clone RTK2758, catalog # 400504) were desalted using Zeba™ Spin Desalting Columns (Thermo Scientific, catalog # 89882). Biotin-CD150 or biotin-IgG antibody was mixed with strep-SAP (Advanced Targeting Systems, catalog # IT-27-1000) in 1:1 molar ratio and then diluted in PBS to desired concentration. CD150-SAP were added to HSC culture medium or administered in 300 μL via tail vein intravenous injection.

In vitro cell viability assay

HSCs were purified and cultured as previously described[1]. HSCs were plated in 96-well plates with 300-500 cells/well in 100 μL volume of cell culture media containing various concentrations of antibody-Saporin-conjugate. After 72 h, cell viability was determined using the Cell Titer assay (Promega, catalog # G9241).

LT-HSC sorting and bone marrow analysis

For LT-HSC sorting, bone marrow (BM) cells were collected by crushing tibias, femurs, pelvic bone and spine (spinal cord was removed). The resulting cell suspension was filtered through 70 μm cell strainers and red blood cells were lysed with red blood cell lysis buffer (ebioscience, catalog #00-4333-57). To improve the sorting efficiency, filtered cells were further enriched by removing lineage positive cells. For details, the filtered cells were first stained with biotin-

conjugated antibodies against lineage markers (CD4, CD8, Gr-1, CD11b, CD5, B220 and Ter119). Lineage positive cells were removed by streptavidin-conjugated magnetic beads (STEMCELL, catalog #19856), and the lineage negative cells were further stained with antibodies against mouse c-Kit, Sca-1, CD48, CD34, CD150. The dead cells were labeled with DAPI. The samples were analyzed by FACS (BD FICR canto-II). HSCs were defined as Lin^{-}Kit^{+}Sca1^{+}Flt3^{-}CD150^{+}CD48^{-}.

For bone marrow cell compartment analysis, bone marrow (BM) cells were collected by crushing tibias and femurs. Red blood cells were removed as described above. The following combinations of cell surface markers were used to define specific cell populations[2]. LT-HSC: Lin^{-}Kit^{+}Sca1^{+}Flt3^{-}CD150^{+}CD48^{-}. MPP1/ST-HSC: Lin^{-}Kit^{+}Sca1^{+}Flt3^{-}CD150^{-}CD48^{-}; MPP2: Lin^{-}Kit^{+}Sca1^{+} Flt3^{-}CD150^{+}CD48^{+}; MPP3: Lin^{-}Kit^{+}Sca1^{+}Flt3^{-}CD150^{-}CD48^{+}; MPP4: Lin^{-}Kit^{+}Sca1^{+}Flt3^{+}CD150^{-}CD48^{+}; CLP: Lin^{-}Kit^{low}Sca1^{low}Flt3^{+}CD127^{+}. MkP: Lin^{-}Kit^{+}Sca1^{-}CD41^{+}CD150^{+}, MEP: Lin^{-}Kit^{+}Sca1^{-}CD41^{-}CD34^{-}CD16/32^{-}, CMP: Lin^{-}Kit^{+}Sca1^{-}CD41^{-}CD34^{+}CD16/32^{-}, GMP: Lin^{-}Kit^{+}Sca1^{-}CD41^{-}CD34^{+}CD16/32^{+}. Erythroblast: FSC^{high}Ter119^{+}CD71^{+}; granulocyte: Ly6G^{+}7/4^{+}B220^{-}Ter119^{-}; monocyte: Ly6G^{-}7/4^{+}B220^{-}Ter119^{-}; pro-/pre-B cells: 7/4^{-}B220^{+}CD127^{+}Ter119^{-}.

Peripheral blood analysis

Up to 30 µl vein blood was collected from retro orbital sinus or tail tips into EDTA-coated tubes. Red blood cells were firstly removed by red blood cell lysis buffer (ebioscience, catalog #00-4333-57). The remaining white blood cells were stained with mixed monoclonal conjugated antibodies (CD45.1, CD45.2, CD3, B220, Gr-1 and CD11b) in FACS staining buffer (PBS containing 1% FBS and 1mM EDTA). After incubation at 4°C in dark for 30 min, samples were washed with FACS staining buffer and resuspended with staining buffer containing 1 µg/ml DAPI. Peripheral blood samples were analyzed by FACS (BD FICR canto-II).

Hematoxylin and eosin (H&E) staining

Mice were sacrificed 3 weeks post-treatment. Tissues including femur, brain, spleen, liver, kidney, heart and lung were collected, fixed in 10% Formalin solution (VWR) and delivered to the Harvard Medical Area Core Specialized Histopathology Services. Hematoxylin and eosin (H&E) staining was performed using standard methods.

Serum biochemical analysis

Blood samples were collected, clotted for 2 h at room temperature, and then centrifuged (1000× g, 10 min) to obtain serum. Serum ALT, AST, Uric Acid, Urea, and Creatinine were analyzed using Cobas Clinical Analyzer (Roche Diagnostics, Indianapolis, IN, USA) at Metabolic Disease Research Center of the University of Massachusetts Chan Medical School.

Complete blood count analysis

For whole mouse blood analysis, 75 µl venous blood was collected from retro orbital sinus and mixed with 225 µl 5mM EDTA. Freshly collected whole blood was analyzed by Hematology system (ADVIA 120) within 2 hours of collection.

References

1. Wilkinson, A. C., Ishida, R., Nakauchi, H. & Yamazaki, S. *Nat Protoc* **15**, 628-648 (2020).
2. Rodriguez-Fraticelli, A. E. *et al. Nature* **553**, 212-216 (2018).
3. Gulati, G. S. *et al. Proceedings of the National Academy of Sciences of the United States of America* **116**, 25115-25125 (2019).

Supplementary Figures

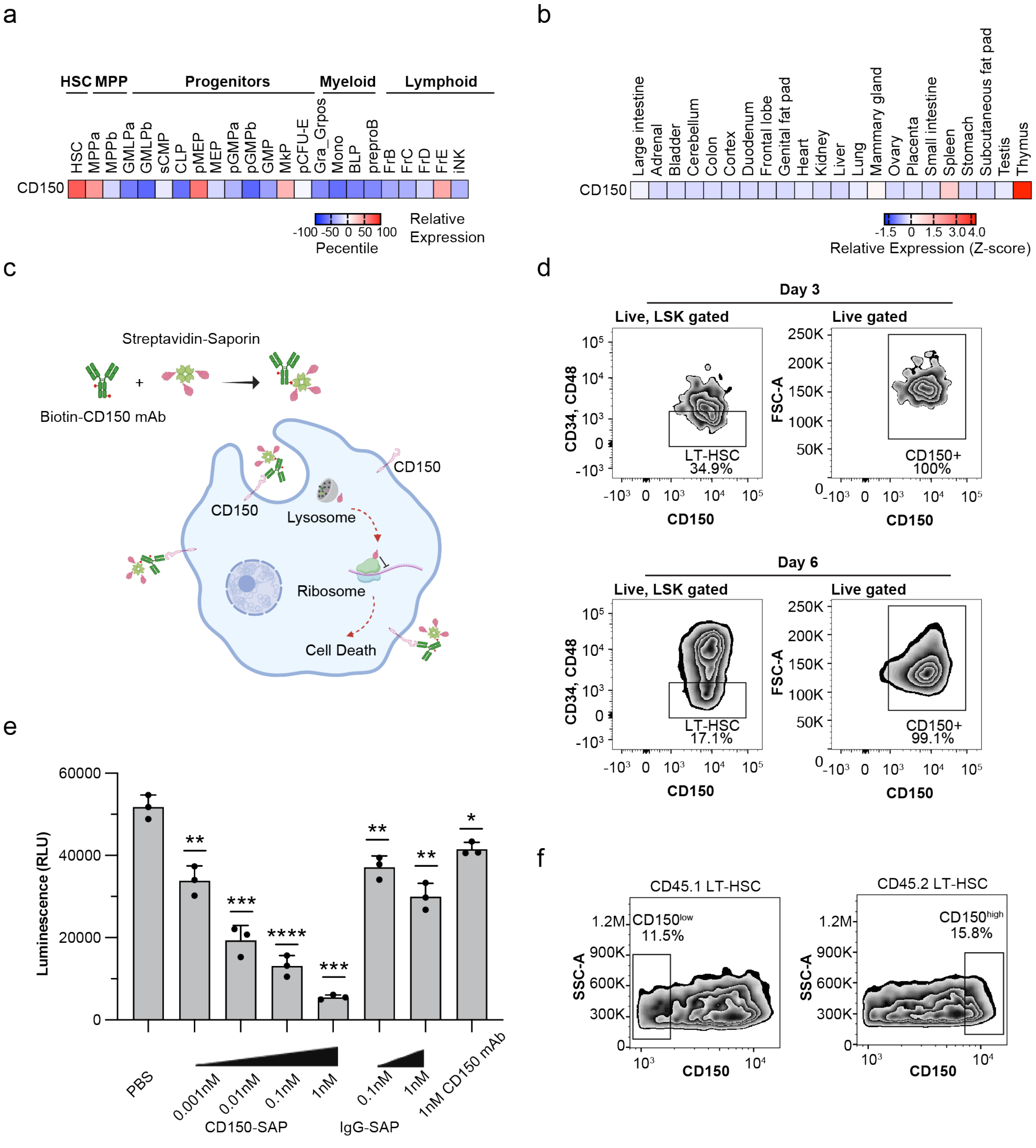

Supplementary Fig. S1 | **Targeted depletion of the dysfunctional CD150^{high} HSCs in vitro.** **a**, Relative RNA expression of CD150 in HSCs, MPPs, progenitors, myeloid, and lymphoid cells. Data for 23 cell types were sourced from Supplementary Table 1 of a previous publication[3]. **b**, Relative RNA expression of CD150 among different tissues and organs. Data is

retrieved from NCBI. RPKM values were z-score normalized for each gene across all tissues. **c**, Diagram illustration of the CD150-SAP mediated cell elimination principle. The diagram was created with BioRender. **d**, Percentage of LT-HSCs and CD150 expression levels following in vitro culture. Top: 3-day culture; Bottom: 6-day culture. LSK: $Lin^{-}Sca1^{+}Kit^{+}$. **e**, Dosage-dependent killing of cultured HSCs in vitro by CD150-SAP, measured by the cell titer assay. **f**, Gating strategy for isolating $CD150^{low}$ and $CD150^{high}$ LT-HSCs from the 17-month-old B6.SJL-*Ptprc*^{a} *Pepc*^{b}/BoyJ (CD45.1) mouse and the 17-month-old C57BL/6J (CD45.2) mouse, respectively. Data are presented as mean ± SD. Statistical significance was determined using One-way ANOVA analysis (e). (* $P < 0.05$, ** $P < 0.01$, *** $P < 0.001$, **** $P < 0.0001$).

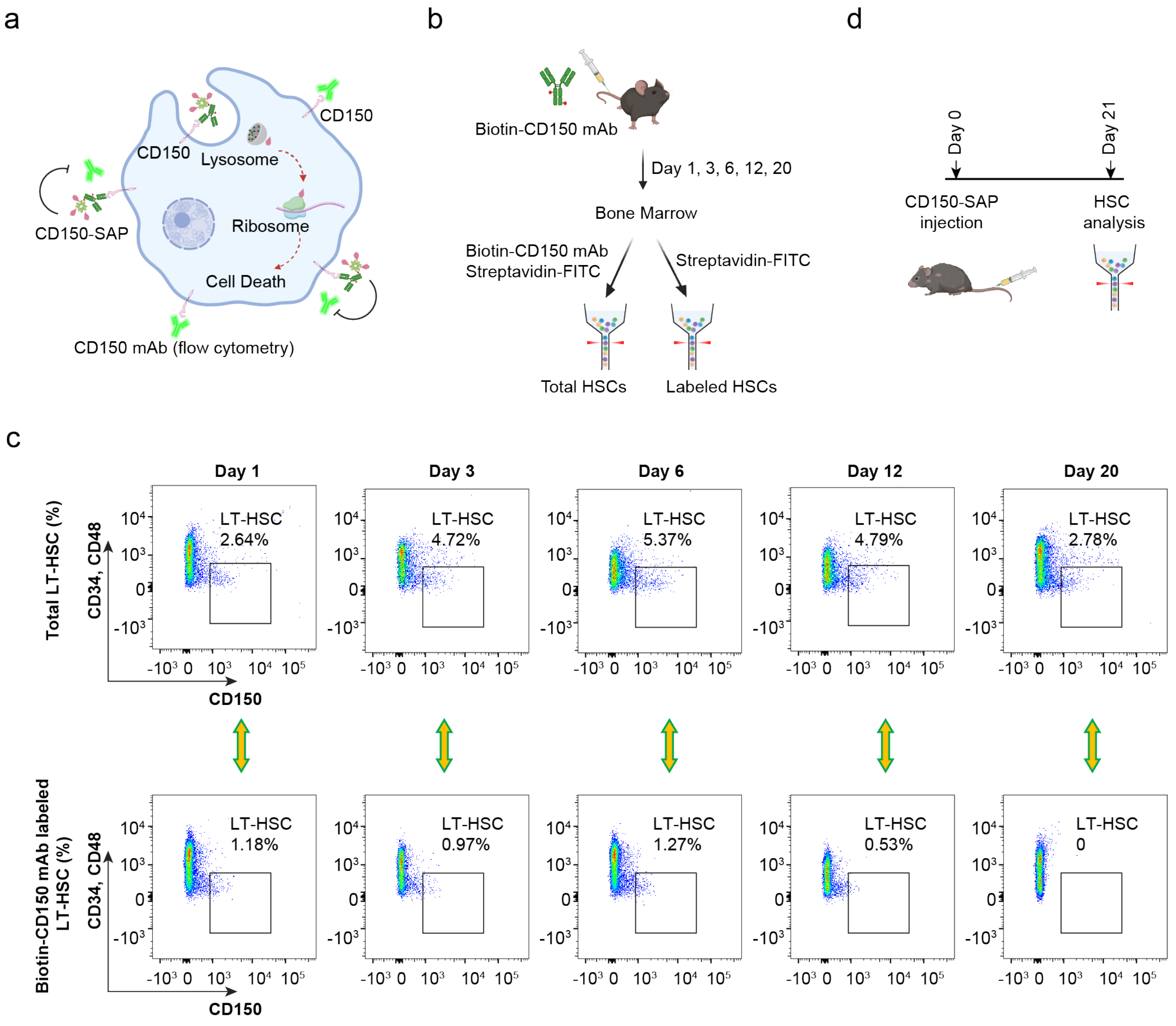

Supplementary Fig. S2 | **Targeted depletion of the dysfunctional CD150^{high} HSCs in vivo**. **a**, Schematic illustration of possible interference of remaining CD150-SAP with CD150 antibody binding in flow cytometry analysis. The diagram was created with BioRender. **b**, Experimental scheme for assessing the in vivo persistency and labeling efficiency of biotin-CD150 antibody on HSCs. Mice were injected with 2.5 mg/kg biotin-CD150 monoclonal antibody. At 1, 3, 6, 12, 20 days, mice were sacrificed, and the CD150 signal on bone marrow derived HSCs was measured by streptavidin-FITC (streptavidin-conjugated FITC). Biotin-CD150 antibody was added to half of the sample to label the total HSCs in the bone marrow, while the other part with no additional biotin-CD150 antibody will be used for HSC labeling by the injected biotin-CD150 antibody. c-Kit, Sca-1, CD48, CD34 antibodies were added to both groups to facilitate the

characterization of LT-HSCs. The diagram was created with BioRender. **c**, Flow cytometry plots showing the percentage of total LT-HSCs (top) and biotin-CD150 monoclonal antibody-labeled LT-HSCs (bottom) at different time points post biotin-CD150 monoclonal antibody injection. **d**, The experimental schedule for CD150-SAP testing in vivo. The diagram was created with BioRender.

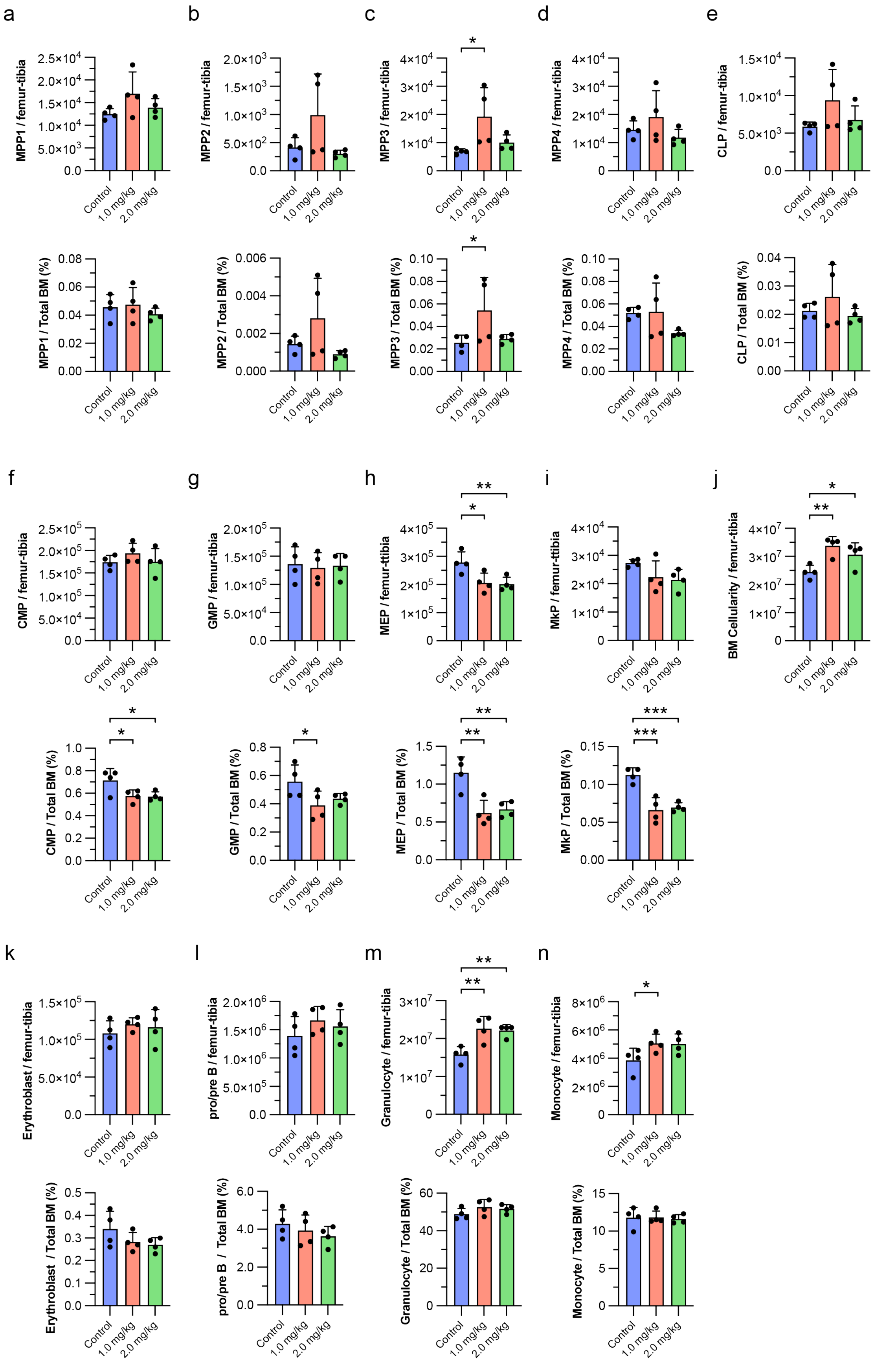
a
b
c
d
e
MPP1 / femur-tibia
MPP2 / femur-tibia
MPP3 / femur-tibia
MPP4 / femur-tibia
CLP / femur-tibia
MPP1 / Total BM (%)
MPP2 / Total BM (%)
MPP3 / Total BM (%)
MPP4 / Total BM (%)
CLP / Total BM (%)
f
g
h
i
j
CMP / femur-tibia
GMP / femur-tibia
MEP / femur-tibia
MkP / femur-tibia
BM Cellularity / femur-tibia
CMP / Total BM (%)
GMP / Total BM (%)
MEP / Total BM (%)
MkP / Total BM (%)
k
l
m
n
Erythroblast / femur-tibia
pro/pre B / femur-tibia
Granulocyte / femur-tibia
Monocyte / femur-tibia
Erythroblast / Total BM (%)
pro/pre B / Total BM (%)
Granulocyte / Total BM (%)
Monocyte / Total BM (%)
Control
1.0 mg/kg
2.0 mg/kg

Supplementary Fig. S3 | **Bone marrow cell compartment analysis. a-i** and **k-n**, Absolute numbers (top) and percentages (bottom) of the indicated bone marrow cell populations three weeks post-treatment. **j**, The bone marrow cellularity of mice 3 weeks post-treatment. Mouse number per group: $n = 4$.

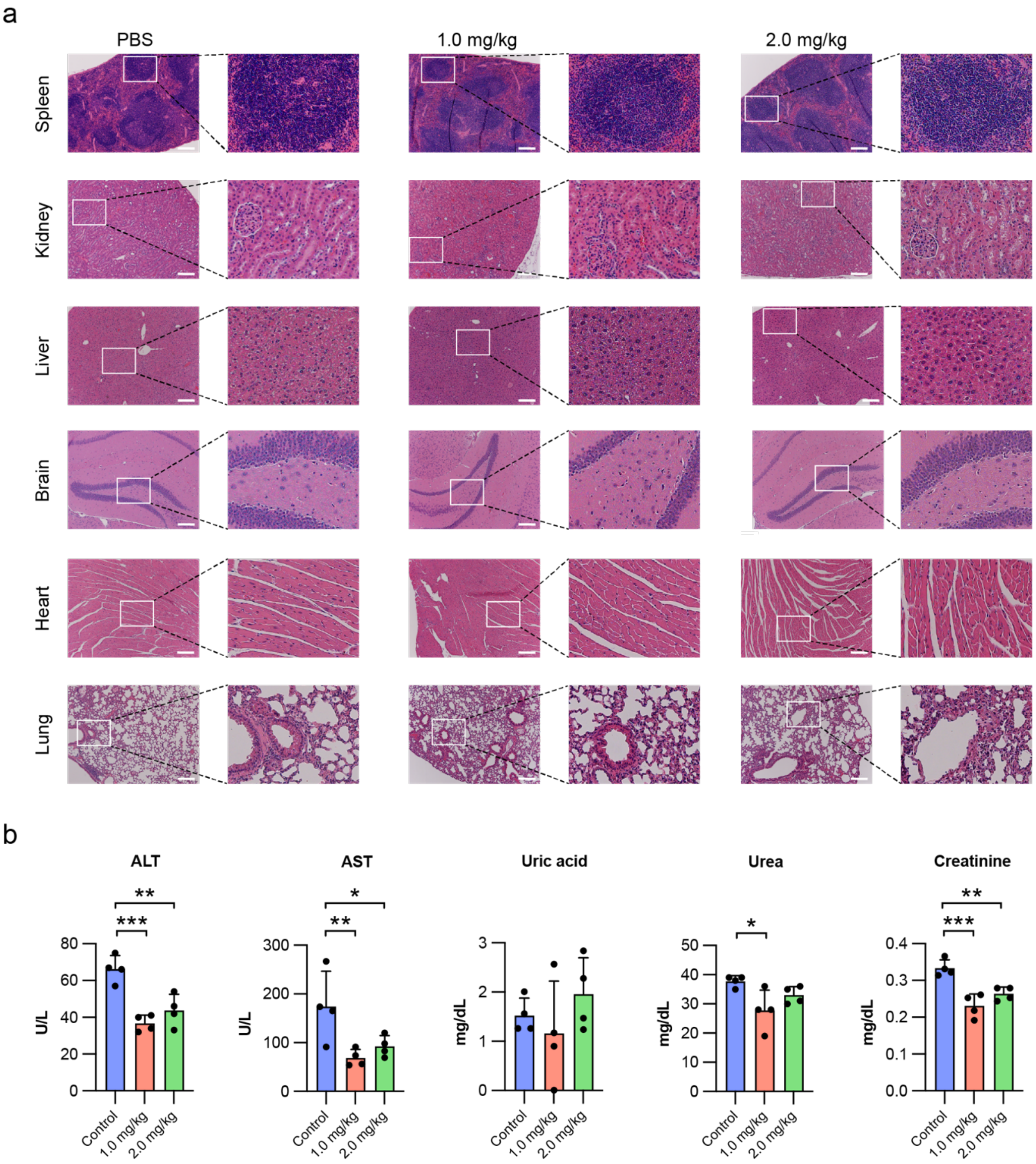

Supplementary Fig. S4 | **Toxicity assessment 3 weeks post CD150-SAP treatment. a**, H&E histology of major organs including spleen, kidney, liver, brain, heart and lung. Scale bar, 20 μm. **b**, Biochemical analysis of liver and kidney function. Liver toxicity was assessed by measuring transaminase levels: alanine aminotransferase (ALT) and aspartate aminotransferase (AST). Kidney toxicity was evaluated by measuring uric acid, urea, and creatinine levels. Mouse number per group: $n = 4$.
